# Scale‐dependent home range optimality for a solitary omnivore

**DOI:** 10.1002/ece3.4690

**Published:** 2018-11-23

**Authors:** Mariela Gantchoff, Guiming Wang, Dean Beyer, Jerrold Belant

**Affiliations:** ^1^ Camp Fire Program in Wildlife Conservation, College of Environmental Science and Forestry State University of New York Syracuse New York; ^2^ Department of Wildlife, Fisheries and Aquaculture Mississippi State University Mississippi State Mississippi; ^3^ Wildlife Division Michigan DNR Marquette Michigan

**Keywords:** carnivore, forest, fragmentation, movement, North America, productivity, space use, ursid

## Abstract

Spatial and temporal heterogeneity are fundamental mechanisms structuring home ranges. Under optimality, an individual should structure their space use economically to maximize fitness. We evaluated support for three hypotheses related to range optimality in American black bears (*Ursus americanus*), predicting (a) range location on a landscape will correspond with high vegetation productivity, (b) increasing forest fragmentation will result in larger ranges, and (c) increasing proportion of forest and/or mean vegetation productivity will result in smaller ranges. We used black bear radio telemetry data from Michigan (2009–2015), Missouri (2010–2016), and Mississippi (2008–2017), USA. Annual space use excluded winter, and we separated seasonal space use into spring, summer, and fall. We collected data from 143 bears (80 females, 63 males), resulting in 97 annual and 538 seasonal ranges. We used generalized linear mixed models to evaluate productivity (estimated through Normalized Difference Vegetation Index [NDVI]) selection, and range size (km^2^) variation between individuals. At the annual scale, black bears consistently selected areas with greater vegetation productivity than the surrounding landscape; yet selection weakened and was more variable seasonally. Opposite to our prediction, we found that increasing fragmentation consistently resulted in smaller ranges; non‐forested land covers and forest edges might provide greater abundance or more diverse foods for bears. Ranges with a greater proportion of forest were smaller, likely reflecting an increase in food and cover which could reduce movements, yet there was no support for more productive ranges also being smaller as expected from an area minimizing strategy. Black bears displayed a scale‐dependent space use strategy: at larger spatial and temporal scales, productivity acted as the strongest limiting factor and energy maximizing was the dominant strategy, while an area minimizing strategy was exhibited seasonally. We revealed consistent, scale‐dependent responses by black bears to environmental conditions, demonstrating the intrinsic plasticity of this adaptable omnivore.

## 1 INTRODUCTION

Movement is a key factor for the survival of most animals and is subject to strong selective pressures (Nathan, 2008; Powell & Mitchell, 2012). Consequently, natural selection should favor movement strategies that maximize fitness, which may manifest as maximized rates of resource acquisition or production of offspring (Austin, Bowen, & McMillan, 2004). Home ranges are the result of animals moving in relation to the distribution of resources (Börger, Dalziel, & Fryxell, 2008; Van Moorter, Rolandsen, Basille, & Gaillard, 2016) at multiple scales (Johnson, 1980 ) and are limited by species traits and evolution (McLoughlin & Ferguson, 2000). Under the framework of optimality, home range location and size should be the result of an individual attempting to structure their space use economically to maximize fitness (Mitchell & Powell, 2004). Mitchel and Powell (2007) proposed two main strategies for an individual optimally selecting spatially heterogeneous resource patches: area minimizing or energy maximizing (similar to time minimizing and rate maximizing; Krebs & Kacelnik, 1991).

The structure and location of mammalian and avian home ranges have been found to be influenced by the spatial distribution of resources (Eide, Jepsen, & Prestrud, 2004; Johnson, Kays, Blackwell, & Macdonald, 2002; Marable, Belant, Godwin, & Wang, 2012; McClintic, Taylor, Jones, Singleton, & Wang, 2014). In particular, home range location (second‐level habitat selection; Johnson, 1980) can be influenced by features such as vegetation type, land use, plant productivity, and risk avoidance (Marchand et al., 2015). Within populations, food availability is likely the main determinant of home range size (McLoughlin & Ferguson, 2000), and size should decrease when high predictability of resource distribution across the landscape allows animals to locate their home range in areas of higher than average quality (Mitchell & Powell, 2004). As productivity increases, individuals following the area minimizing strategy should need a smaller area to fulfill their energetic needs, therefore, moving shorter distances and displaying smaller home ranges (Barraquand & Murrell, 2012; Dahle & Swenson, 2003; McNab, 1963). In addition, the patchiness of resources on the landscape can further structure home ranges areas. The resource dispersion hypothesis, originally a hypothesis of mammalian gregariousness (Carr & Macdonald, 1986), states that as spatial variability of resources increases, individuals must use a larger area to acquire sufficient resources (Macdonald & Johnson, 2015). For an individual following an area minimizing strategy, increased dispersion of resources should result in a larger home range. Resource dispersion can occur naturally in heterogeneous landscapes, or be a result of anthropogenic habitat fragmentation; mammals of all sizes have experienced habitat loss and fragmentation around the world (Crooks, 2002; Crooks et al., 2017), affecting their space use and behavior (Tucker et al., 2018; Wolf & Ripple, 2017).

Studying how species modify their space use depending on landscape structure is vital for managing species such as American black bears (*Ursus americanus*), which are currently recolonizing parts of North America after major past range contractions (Scheick & McCown, 2014) and displaying increased conflict in human‐modified areas (Baruch‐Mordo, Breck, Wilson, & Theobald, 2008; McFadden–Hiller, Beyer, & Belant, 2016). Our objective was to evaluate black bear optimality regarding annual and seasonal home range location and size (Table 1). Black bears are very suitable for testing hypotheses of home range optimization because they display site fidelity, use heterogeneous habitats, and their food resources are mostly fixed in space (i.e., vegetation; Mitchell & Powell, 2007). We analyzed if home range location on the landscape is influenced by vegetation productivity, and examined the influence of extrinsic factors on home range size (Table 1). Within this conceptual framework, bears behaving optimally should display home ranges that are, on average, more productive than the study area, and seasonal home ranges should be more productive compared to the area around them, reflecting economically driven behavior. In addition, bears are forest obligate species (Herrero, 1972; Pelton, 2003) and individuals following an area minimizing strategy should display smaller home ranges as vegetation productivity and forest proportion increase, and spatial variability of resources (fragmentation) decreases.

**Table 1:** Hypotheses evaluating optimality in black bear home range location and size, together with associated factors, predictions, and support

Hypothesis	Predictions
Food selection	Home ranges will be more productive than surrounding areas
Fragmentation	Greater edge density results in larger home ranges
Productivity	Greater forest proportion results in smaller home ranges
	Greater productivity results in smaller home ranges

## 2 METHODS

### 2.1 Study areas

**Figure 1:**
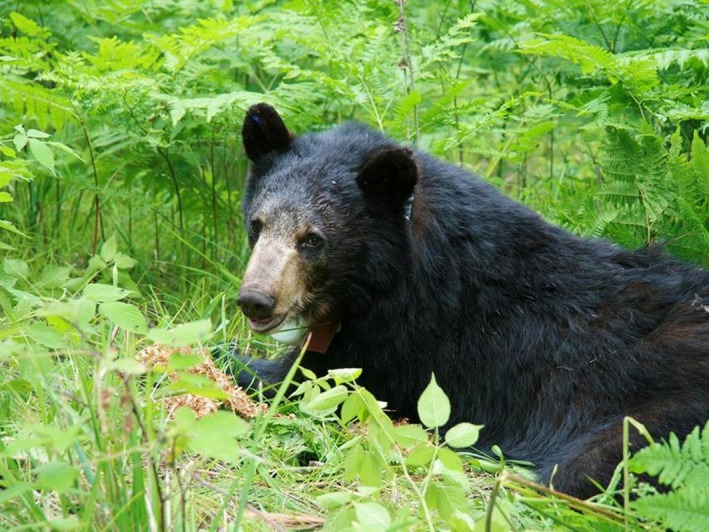
Collared American black bear (*Ursus americanus*) in Michigan, USA

**Figure 2:**
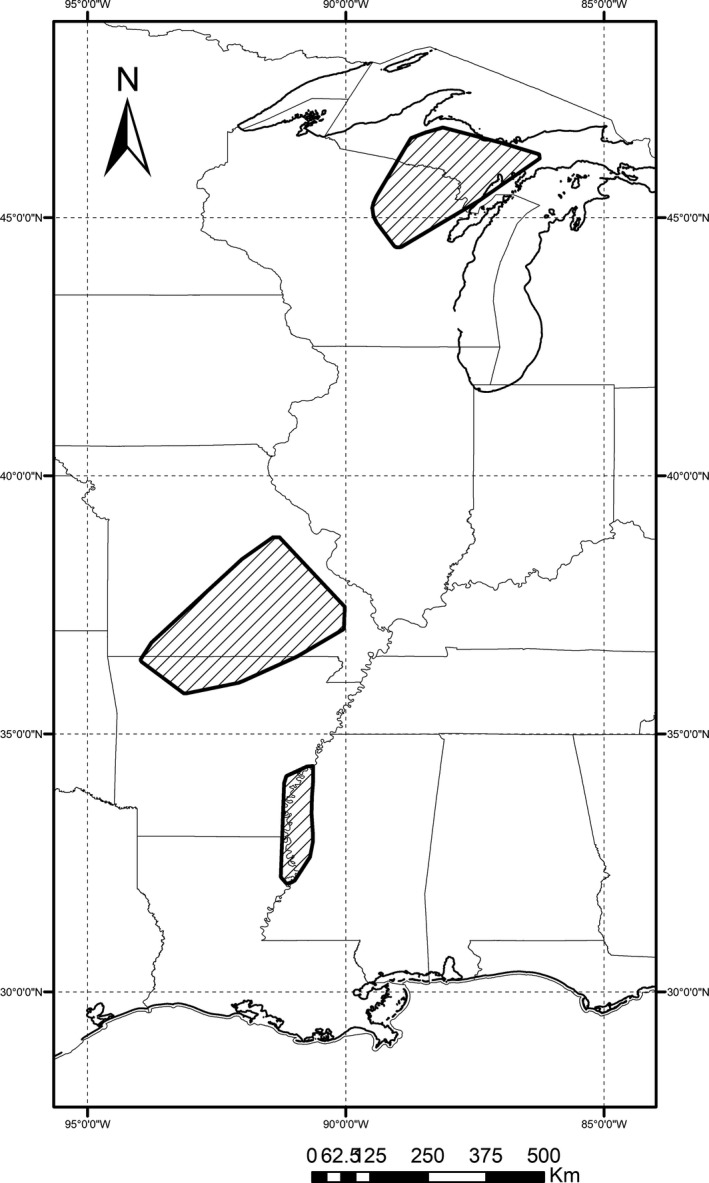
Location of the three black bear study areas (dashed polygons) located primarily in Michigan (top), Missouri (middle), and Mississippi (bottom), USA

We used data from black bear (Figure 1) studies in Michigan (MI, data from 2009 to 2011 and 2013 to 2015), Missouri (MO, data from 2010 to 2016), and Mississippi (MS, data from 2008 to 2017), USA (Figure 2). In MS, topography is generally flat with elevations from 0 to 247 m above sea level. Vegetation is primarily agricultural land with forested areas along the Mississippi River. Agricultural and urban lands comprise about 45,000 and 2,400 km^2^ of the state, respectively (Mississippi Automated Resource Information System 2014). Bear density throughout MS was estimated at <1/100 km^2^ (R. Rummell, Mississippi Department of Wildlife, Fisheries, and Parks [MDWFP], pers. comm.). Black bears were captured in the Delta region of western MS, where most black bear sightings occur (Simek, Belant, Young, Shropshire, & Leopold, 2012). In MO, data collection was conducted in the Ozark Highlands. This region contains karst topography with elevations from about 70 to 280 m and has a humid warm continental climate. Dominant land covers include forest, crop and pasture, grassland, and human developed areas (Karstensen, 2010), and black bear density was 1.7/100 km^2^ (Wilton et al., 2014). In MI, data collection was conducted in the Upper Peninsula. This area has flat topography with elevations ranging approximately from 160 to 240 m and a humid cold continental climate. Predominant vegetation includes upland and lowland hardwoods, lowland conifer swamps, upland conifers, aspen (*Populus* spp.) stands, row‐crop, and livestock agriculture, and some herbaceous wetlands (Duquette, Belant, Svoboda, Beyer, & Lederle, 2014). Black bear density is 14–19/100 km^2^ (J. L. Belant, unpublished data). Black bears in MS and MO are not harvested, and in MI they are harvested annually during September and October; only males and females without dependent young are legal for harvest (Belant, Etter, Mayhew, Visser, & Friedrich, 2011).

### 2.2 Capture and marking

Black bears on each study area were captured using modified Aldrich foot snares (Johnson & Pelton, 1980) and culvert traps. Captured individuals were immobilized with tiletamine and zolazepam at a dosage of 4–7 mg/kg of estimated body weight (Telazol; A. H. Robins Company, Richmond, Virginia, USA), administered with a syringe pole or dart syringe fired from a CO_2_‐powered pistol or rifle. Each bear received a GPS radiocollar: MI (Lotek Wireless, Newmarket, Ontario, Canada), MO (Northstar RASSL Globalstar, King George, Virginia, USA; Advanced Telemetry Systems M2610B, Isanti, Minnesota, USA; Lotek Wireless 7000MU, Newmarket, Ontario, Canada), MS (Telonics Inc., Mesa, Arizona, USA; Lotek Iridium Collars, Lotek Wireless Inc., Newmarket, Ontario, Canada). All collars had leather breakaway links (Garshelis & McLaughlin, 1998). All capturing and handling of bears follow the American Society of Mammalogists guidelines (Sikes & Gannon, 2011) and was approved by the Mississippi State University Institutional Animal Care and Use Committee (MS protocol: 14‐098, MO: 13‐094, MI: 15‐013). We located dens using aerial and ground‐based telemetry, and we recovered data from GPS collars during recaptures, UHF data downloads during flights, and den visits.

### 2.3 Data Analysis

For home range (hereafter range) analyses we randomly subsampled location estimates such that no individual had >1 location per day, reducing temporal autocorrelation and standardizing relocation intervals among data sets (Hiller, Belant, & Beringer, 2015). To describe annual space use, we separated data by year and excluded data collected during the denning period. Annual activity includes locations from 15 March to 30 November for MS and MO and 15 April to 31 October for MI. To describe seasonal space use, we separated data into three seasons: spring (den emergence; 15 March to 31 May in MS and MO, 15 April to 15 June in MI), summer (mating and dispersal; 1 June to 31 August in MS and MO, 15 June to 31 August in MI), and fall (hyperphagia; 1 September to 30 November in MS and MO, 1 September to 31 October in MI), (Benson & Chamberlain, 2006, Hiller et al., 2015, J. L. Belant unpublished data). To estimate space use, we used fixed‐kernel techniques with plug‐in bandwidths (Gitzen, Millspaugh, & Kernohan, 2006) to determine the area of the 95% utilization distribution (UD) within a given range (Kernohan, Gitzen, & Millspaugh, 2001) using the *rhr* package in program R (R Core Team, 2013). The plug‐in method minimizes oversmoothing in resulting kernels generated from GPS data (Kertson & Marzluff, 2011). Cell size for kernel smoothing was kept constant among all home range calculations to allow for direct comparisons of range size. We considered data adequate for range modeling when locations spanned at least 75% of the time period being analyzed (e.g., ≥68 of 90 days covered), and exceeded 40 and 25 relocations for annual and seasonal range estimation (Börger et al., 2006; Haines, Hernandez, Henke, & Bingham, 2009; Powell, 2000). For individuals with data for >1 year or >1 season, each seasonal or annual range from each bear was considered a sampling unit.

Black bears throughout their range have an opportunistic omnivorous diet dominated by plants and insects (Pelton, 2003, Costello et al. 2016). To assess if range location within the study area was influenced by environmental productivity, we used the Normalized Difference Vegetation Index (NDVI) to measure vegetation greenness as an index of plant productivity. The relationship between NDVI and average energy availability is well established (Pettorelli et al., 2006; Wiegand, Naves, Garbulsky, & Fernández, 2008), and has been employed for taxa including herbivores (Garel et al., 2006) and brown bears (Bojarska & Selva, 2012). We used the 16‐day composite NDVI data from the eMODIS server (250‐m resolution; United States Geological Survey). We rescaled raw NDVI values by multiplying them by a factor of 0.0001. We calculated the average NDVI of the annual range for each bear in each study site and each year and calculated the average NDVI for each study area and each year. The study area was determined as the minimum convex polygon that included all ranges in each state. To assess seasonal selection, we calculated the mean NDVI of each seasonal range for each bear and year (spring, summer, fall), and compared it to the NDVI for that same time period for a buffer surrounding the seasonal range, representing potential movement in a 2–3 month period. To obtain the buffer distance, we calculated the average seasonal range size for females and males separately and used the respective radius to create the buffer around ranges.

To assess spatial configuration of forested land covers, we used 30‐m resolution data from the U.S. Geological Survey (Homer et al., 2015). We designated all forested land covers and woody wetlands (NLCD 41, 42, 43, 90) as potential black bear habitat (Sollmann, Gardner, Belant, Wilton, & Beringer, 2016), and calculated proportion of forest and forest patch edge density for each range using the package SDMTools in R v.3.3.2 (R Development Core Team, 2013).

We used generalized linear mixed models (GLMM) with a Gaussian distribution to assess variation in productivity (NDVI) and area (km^2^) of annual or seasonal ranges of individual bears in relation to individual, site, temporal, and environmental covariates. We chose four analyses to evaluate our hypotheses (Table 2), selecting ecologically relevant factors and interactions. We included bear ID and year as potential random effects in all analyses. We used Pearson's correlation coefficient (*r*) to test for multicollinearity among independent variables. If |*r*| < 0.70 for any pair of independent variables (Dormann et al., 2013), we assumed multicollinearity did not compromise models results. If multicollinearity existed for a pair of variables, they were not included in the same model. To assess whether data were normally distributed, we examined residuals for indication of systematic lack of fit using the global model and full data set. When the response variable had a skewed distribution (i.e., area), we transformed the data (log_10_) to increase homogeneity of the variance. Factors were scaled before analysis to allow comparisons of effects. We used package *lme4* in R (R Development Core Team, 2013) to run the GLMMs. We used Akaike Information Criterion adjusted for small samples (AICc) to rank models based on model complexity and fit (Burnham & Anderson, 2002). For each analysis, (Table 2) we first performed all combinations of random factors (plus all fixed effects) to find the best fitting random structure (lowest AICc score) for the data. We then performed all combinations of selected fixed factors and interactions (Table 2), always including the chosen random structure. All models within an AICc difference of <2 from the top‐ranked model were considered top models. To avoid overparameterization, we chose the simplest model (lowest value for degrees of freedom) within top models as the “best fit model”: a compromise between simplicity and explanatory power (Richards, Whittingham, & Stephens, 2011). We also considered *R* ^2^ values to describe performance and compare between top models. We calculated both conditional *R* ^2^ (all variance explained) and marginal *R* ^2^ (variance explained only by fixed factors) via R Package MuMin (Barton, 2013) based on Nakagawa and Schielzeth (2013). For the best fit model, we estimated parameter coefficients, standard errors, and 95% confidence intervals. We used R v.3.3.2 (R Development Core Team, 2013) for all statistical analyses.

**Table 2:** Selected factors and interactions for analyses of (a) annual (*n* = 97), and (b) seasonal (*n* = 538) productivity selection and (c) annual (*n* = 97) and (d) seasonal (*n* = 538) home range size variation for black bears

	Fixed factors	Interactions	Response
(a)	State	Sex*State	Normalized Difference Vegetation Index (NDVI) difference between annual home range and study area
	Sex		
(b)	Sex	Season*Sex	NDVI difference between seasonal home range and seasonal buffer
	Season	Season*State	
	State	Sex*State	
(c)	Prop forest	Sex*State	Size of annual home range
	Edge density		
	Mean NDVI		
	State		
	Sex		
(d)	Prop forest	Season*Sex	Size of seasonal home range
	Edge density	Season*State	
	Mean NDVI	Sex*State	
	Season		
	State		
	Sex		

## 3 RESULTS

We collected data adequate for range modeling from 143 bears; 43 from MI (19 F, 24 M), 73 from MO (45 F, 28 M), and 27 from MS (16 F, 11 M). Range sizes are presented as median values as data were non‐normally distributed. The median annual range area was 18.7 km^2^ for females and 89.9 km^2^ for males (Supporting information Appendix S1 Table A). Male annual ranges were 5.8 times larger than females in MI, 5.3 times larger in MO, and 3.8 times larger in MS. The median seasonal range area for all bears was 15.4 km^2^ for females and 59.3 km^2^ for males (Supporting information Appendix S1 Table A). The median proportion of forest within seasonal ranges was 0.85 in MI (0.87 F, 0.83 M), 0.92 in MO (0.93 F, 0.89 M), and 0.85 in MS (0.89 F, 0.67 M). Seasonal proportion of forest ranged from 0.85 to 0.96 for females, and from 0.67 to 0.92 for males (Supporting information Appendix S1 Table A). Within each of our study areas, the most abundant land covers not considered black bear habitat were cultivated crops in MI and MS, and pasture/hay in MO.

### 3.1 Productivity selection

**Figure 3:**
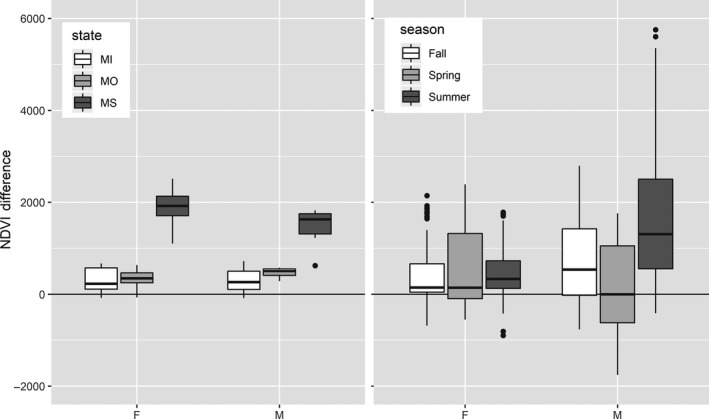
Normalized Difference Vegetation Index (NDVI) difference (rescaled by a factor of 0.0001) for female and male black bear (a) annual home ranges and study area (*n* = 97) and (b) seasonal home ranges and seasonal buffers (*n* = 538), in Michigan (MI), Missouri (MO), and Mississippi (MS), USA. Positive values indicate selection and negative values avoidance

The selected random structure for productivity selection at the annual scale was bear ID and year. One model best described the annual NDVI difference (Table 2, analysis A), which contained all fixed factors and one interaction (Table 3), with an AICc weight of 0.99. Conditional *R* ^2^ was 0.93 and marginal *R* ^2^ was 0.86. There were no competing models. Bears in all states selected areas with greater NDVI than the study areas (Table 3, Figure 3). There was no difference in selection between MI and MO, and bears in MS showed stronger selection than the other two states. Males in MS selected areas of lower NDVI than the female–male difference in MI and MO.

**Table 3:** Parameter estimates and standard deviations (*SD*) for annual home range productivity selection (Normalized Difference Vegetation Index difference) for black bears (2008–2017) in Michigan (MI), Missouri (MO), and Mississippi (MS), USA

Parameter	Estimate	*SD*
Intercept	0.31	0.09*
State MO	0.10	0.09
State MS	1.67	0.09*
Sex M	−0.01	0.12
State MO: sex M	0.02	0.18
State MS: sex M	−0.63	0.18*

*p* < 0.05.

The selected random structure for productivity selection at the seasonal scale was bear ID and year. One model best described the NDVI difference between seasonal ranges and seasonal buffers (Table 2, analysis B) which consisted of all fixed factors and interactions (Table 4), with an AICc weight of 0.99. Conditional *R* ^2^ was 0.64 and marginal *R* ^2^ was 0.42. There were no competing models. There was selection for greater productivity in summer ranges by both sexes (Table 4, Figure 3). Several interactions revealed more detailed patterns, for example, male bears use areas less productive than surrounding buffers during spring and areas more productive during summer, and we quantified interesting variations in selection for each study area by season and sex (Table 4).

**Table 4:** Parameter estimates and standard deviations (*SD*) for seasonal home range productivity selection (Normalized Difference Vegetation Index difference) for black bears (2008–2017) in Michigan (MI), Missouri (MO), and Mississippi (MS), USA

Parameter	Estimate	*SD*
Intercept	0.03	0.19
Sex: Male	−0.11	0.21
Season Spring	0.24	0.19
Season Summer	0.38	0.17*
State MO	0.13	0.20
State MS	0.99	0.22*
Sex Male: season Spring	−0.53	0.17*
Sex Male: season Summer	1.06	0.14*
Sex Male: state MO	1.02	0.22*
Sex Male: state MS	−0.93	0.27*
Season Spring: state MO	−0.49	0.21*
Season Summer: state MO	−0.23	0.18
Season Spring: state MS	0.24	0.22
Season Summer: state MS	−0.52	0.19*

*p* < 0.05.

### 3.2 Home range size variation

**Figure 4:**
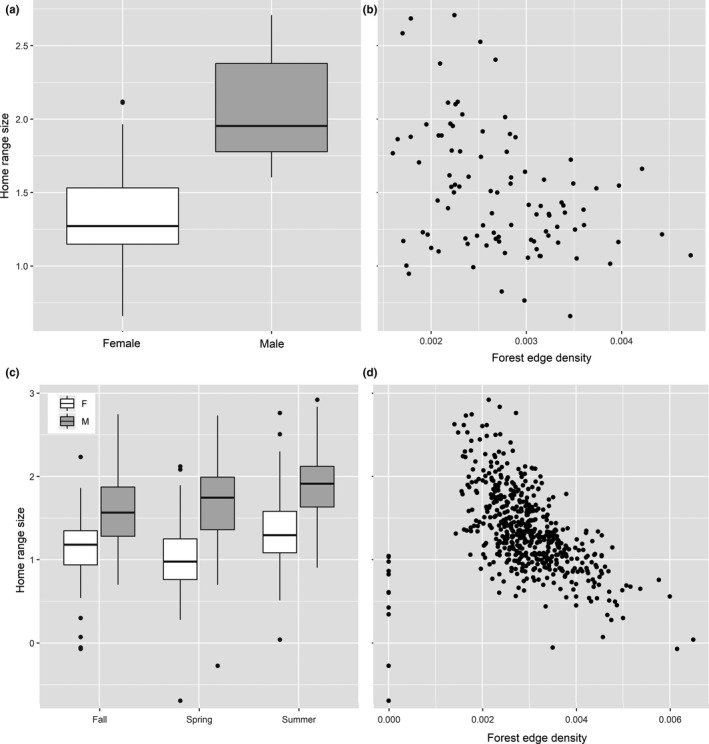
Black bear annual home range sizes (log_10_ transformed, *n* = 97) in relation to (a) sex, and (b) forest edge density. Seasonal home range sizes (log_10_ transformed, *n* = 538) in relation to (c) sex and season, and (d) forest edge density

The selected random structure for annual size variation was bear ID. Three competing models (Supporting information Appendix S1 Table B) best‐described size variation in annual ranges; the best fit model (Table 5) included the effects of sex and forest edge density, with no interactions. This model had a conditional *R* ^2^ of 0.85 and marginal *R* ^2^ was 0.49. Males had larger ranges than females, and forest edge density had a negative influence on annual range area (Table 5, Figure 4).

**Table 5:** Parameter estimates and standard deviations (*SD*) for annual home range size variation for black bears (2008–2017) in Michigan (MI), Missouri (MO), and Mississippi (MS), USA

Parameter	Estimate	*SD*
Intercept	1.37	0.04*
Sex: Male	0.67	0.08*
Forest edge density	−0.10	0.02*

*p* < 0.05.

The selected random structure for seasonal size variation was bear ID. Two competing models (Supporting information Appendix S1 Table C) best described the size variation in seasonal ranges; the best fit model (Table 6) included sex, state, season, edge density, proportion of forest, and two interactions. This model had a conditional *R* ^2^ of 0.68 and marginal *R* ^2^ of 0.55. Overall, males displayed larger seasonal ranges than females, and summer ranges were largest for both sexes (Table 6, Figure 4). Forest edge density and proportion of forest had a negative influence on range area for all bears, but edge density was three times more influential. Males in the spring and summer had larger ranges than in the fall. Bears in MO had larger ranges, and in MS smaller ranges (spring and summer only), than MI (Table 6, Figure 4).

**Table 6:** Parameter estimates and standard deviations (*SD*) for seasonal home range size variation for black bears (2008–2017) in Michigan (MI), Missouri (MO), and Mississippi (MS), USA

Parameter	Estimate	Std.
(Intercept)	1.11	0.08*
Sex Male	0.35	0.06*
Season Spring	−0.02	0.09
Season Summer	0.21	0.08*
State MO	0.17	0.08*
State MS	−0.01	0.09
Forest edge density	−0.18	0.02*
Proportion of forest	−0.06	0.02*
Season Spring: sex Male	0.20	0.08*
Season Summer: sex Male	0.14	0.07*
Season Spring: state MO	0.09	0.10
Season Summer: state MO	−0.03	0.09
Season Spring: state MS	−0.37	0.10*
Season Summer: state MS	−0.22	0.09*

*p* < 0.05.

## 4 DISCUSSION

As expected for a behaviorally flexible species with a large geographic range, black bears demonstrated high individual variability in spatial selection and range size; nevertheless, we observed broad patterns. Annually, black bears consistently selected areas with greater vegetation productivity than the surrounding landscape; yet selection for productivity weakened and became more variable seasonally. Opposite to our prediction, increasing fragmentation of forest patches consistently resulted in smaller annual and seasonal ranges in all areas. In contrast, our results supported our prediction that ranges with proportionately more forest would be smaller, but found no support for more productive ranges also being smaller as we would expect with an area minimizing strategy.

Under the framework of optimality, home range location should reflect a fitness‐maximizing strategy (Mitchell & Powell, 2004, 2007 ) while achieving nutritional security (Macdonald & Johnson, 2015). Different limiting factors can act at different scales (Rettie & Messier, 2000), and our results suggest vegetation productivity can influence spatial selection at coarser spatial and temporal scales. Different mammalian taxa, such as ungulates (Stillfried et al., 2017), primates (Zinner, Pelaez, & Torkler, 2002), and carnivores (Duquette et al., 2017; Mitchell & Powell, 2007), have been found to use areas of greater quality than the surrounding landscape, yet the limiting effect of vegetation productivity in black bear space use becomes weaker at finer scales reflecting a shift in limiting factors. Similarly, NDVI had little to no explanatory power related to fine scale (3rd or 4th scale; Johnson, 1980) spatial selection for wolves (Milakovic et al., 2011) or black bears (Duquette et al., 2017). Alternatively, NDVI may be limited to serve as an adequate proxy for omnivore food resources at finer scales. NDVI mirrors climatic conditions and can function as a good coarse proxy for overall vegetation productivity and food availability, however, at smaller scales it can fail to reflect the abundance of some food items like fruits, insects, or mast (Bojarska & Selva, 2012; Zhou et al., 2011). In addition, the finest resolution for NDVI layers (250 m) might not provide enough variability for smaller scale analyses if individuals are already selecting areas more productive at larger scales; detecting selection for homogeneous or common resources at certain scales is problematic (Kertson & Marzluff, 2011), that is, detecting selection within selection.

Increasing forest edge density had a consistent negative relationship with black bear range size both annually and seasonally. This relationship was surprising as we predicted forest fragmentation would cause bears to increase movement to obtain enough food resources located within forests. An alternative explanation is that non‐forested land covers and forest edges themselves can be a source of food resources for bears. For example, forest edges often facilitate the growth of a diversity of early successional vegetation that opportunistic omnivores can consume (Litvaitis 2001). Black bears in other areas have been associated with diverse land covers (Carter, Brown, Etter, & Visser, 2010), and reduce their ranges in areas with forest clearcuts (Brodeur, Ouellet, Courtois, & Fortin, 2008). Forest edge density could potentially act as a superior proxy than NDVI for black bear food resources at a fine scale; if so, then the prediction of smaller ranges when more food is available, under an area minimizing approach, would be supported. Human‐derived landscape fragmentation can result in smaller ranges by allowing resource generalists to take advantage of resources in areas surrounding their ranges (Andren, 1994; Tigas, Vuren, & Sauvajot, 2002). Black bears that have regular access to human‐derived food are larger, move less, and use less natural food (Massé, Dussault, Dussault, & Ibarzabal, 2014) and a recent global assessment found that mammals in human‐modified areas have decreased movement rates (Tucker et al., 2018). But the negative relationship between fragmentation and range area can also occur when little to no anthropogenic disturbances occur; for example, brown bears in Alaska used smaller areas as the landscape became more heterogeneous (Mangipane et al., 2018) and larger home range sizes were linked to areas of lower habitat diversity for black bears in Arkansas (Smith and Pelton 1990). In contrast, Karelus, McCown, Scheick, Kerk, and Oli (2016) suggested that fragmentation caused black bears in Florida to use larger areas, and a black bear study in Missouri suggested that increasing land cover diversity resulted in larger range areas (Hiller et al., 2015). Detecting and measuring the effect of landscape fragmentation on species is dependent on the vegetation community being fragmented and what is considered “non‐habitat”; human‐modified land covers can be hostile to some species, or populations within species, while providing attractive resources to others (Crooks, 2002).

Proportion of forest had a negative influence on seasonal range sizes, supporting our prediction of an area minimizing strategy (Mitchell & Powell, 2004, 2007 ). More available forested areas likely reflect an increase in food, water, and shelter which would result in reduced movements. Though black bears are a forest obligate species (Herrero, 1972; Pelton, 2003), they can use non‐forested areas (e.g., agriculture or low‐density human areas) to supplement feeding. In Missouri, black bear density declined with increasing forest cover (Sollmann et al., 2016), and when sufficient forest is available, human‐modified areas can provide attractive food resources (Beckmann & Berger, 2003; Ditmer et al., 2015; Merkle, Robinson, Krausman, & Alaback, 2013). Male bears in this study had lower proportion of forest within their ranges than female conspecifics, possibly reflecting risky food‐seeking behavior, mate seeking, or exploratory movements (Beckmann & Berger, 2003; Merkle et al., 2013). Though we expected more productive ranges (mean NDVI) to be smaller than less productive ranges following an area minimizing strategy, that prediction was not supported; ranges overall had high NDVI values possibly suggesting an energy maximizing approach. A negative relationship between productivity and range size has been observed in other carnivores (Bengsen et al., 2016; Ferguson, Currit, & Weckerly, 2009; Herfindal, Linnell, Odden, Nilsen, & Andersen, 2005), though the form of these relationships has been inconsistent among species (Nilsen, Herfindal, & Linnell, 2005).

In addition to our main predictions, we found that differences in range sizes existed between males and females annually and during all seasons; males displayed ranges from two to six times larger than females. In a polygynous mating system, adult males are expected to structure their space use to maximize mating opportunities (Sandell, 1989) and male range sizes should be greater than required for metabolic requirements (Dahle & Swenson, 2003; Liberg, Sandell, Pontier, & Natoli, 2000; Sandell, 1989); our results support these predictions. In other solitary polygynous carnivores (e.g., bobcats), male range areas are partially determined by female range areas (Ferguson et al., 2009). In agreement, we found that male ranges were largest where female ranges were also the largest (Missouri). Both female and male bears had the largest ranges during the mating and dispersal period (June‐July), consistent with previous studies (e.g., Costello, Creel, Kalinowski, Vu, & Quigley, 2009; Massé et al., 2014). Some of the smallest black bear ranges have been reported in the highly productive areas in the Mississippi Delta (Benson & Chamberlain, 2007; Oli, Jacobson, & Leopold, 2002), which is consistent with our results. We also found that bears in Mississippi had the greatest productivity selection (Table 3) which might be partially influenced by landscape structure; the Mississippi Delta includes highly productive hardwood forests constrained by less productive agriculture. Notably, ranges for males in Michigan during fall were smaller than for males in other areas, possibly related to seasonal risk avoidance in relation to black bear harvest (Stillfried, Belant, Svoboda, Beyer, & Kramer‐Schadt, 2015); while baiting may facilitate those limited movements by providing clustered high energy resources. Finally, increased population density should result in overall smaller ranges on average when compared to less dense populations (Kjellander et al., 2004), yet we did not find a pattern of increasing population density resulting in increasingly smaller ranges for our three study areas.

Black bears did not display a clearly defined scale‐independent strategy for structuring ranges (energy maximizing or area minimizing), consistent with a previous study (Mitchell & Powell, 2007). The high productivity of ranges of all sizes suggests energy maximizing, while the negative relationship between range size and both fragmentation and forest proportion suggests area minimizing. More limiting factors act at larger scales (Rettie & Messier, 2000), which would suggest productivity is the strongest limiting factor and energy maximizing is the dominant strategy while plasticity allows for seasonal area minimizing. The life history of black bears points to them being energy maximizers; species whose potential reproductive success is related to their energy gain (McLoughlin, Ferguson, & Messier, 2000). For many ursids, body mass and body fat have been found to influence reproductive success of males (Costello et al., 2009) and females (Atkinson & Ramsay, 1995; Belant, Kielland, Follmann, & Adams, 2006; López‐Alfaro, Robbins, Zedrosser, & Nielsen, 2013; Robbins, Ben‐David, Fortin, & Nelson, 2012), and their typical weight fluctuations during the year (i.e., hyperphagia, hibernation, den emergence; Hellgren, 1998; Farley & Robbins, 1995) should be facilitated by an energy maximizing strategy. In addition, the usual lack of territoriality (Mitchell & Powell, 2007) would facilitate an “expansionist” or energy maximizer behavior (Macdonald & Johnson, 2015) and dietary studies on captive and wild black bears suggest they fit an energy maximizing strategy (Costello et al. 2016).

Spatial and temporal heterogeneity are fundamental mechanisms structuring ranges (Börger et al., 2008; Macdonald & Johnson, 2015; Mitchell & Powell, 2007) and will become increasingly important as human modification of the landscape continues to influence species’ movements (Tucker et al., 2018). Black bears optimally locate their annual ranges to maximize access to areas of high vegetation productivity while adapting their space use to the amount of forest available and the degree of fragmentation, displaying scale‐dependent energy maximizing and area minimizing strategies. By quantifying black bear space use across different areas, over time, and among and within individuals, we revealed consistent large‐scale responses to environmental conditions while highlighting the intrinsic plasticity of this flexible omnivore.

## CONFLICT OF INTEREST

None declared.

## AUTHOR CONTRIBUTIONS

MG and JB conceptualized and designed the study. MG, DB, and JB acquired the data. MG, GW, and JB analyzed and interpreted the data. MG and JB drafted the manuscript. MG, JB, GW, and DB provided critical revisions.

## DATA ACCESSIBILITY

Data used in this manuscript is accessible in Dryad, https://doi.org/10.5061/dryad.3vc108b.
