# Supplementary material for: Scale‐dependent home range optimality for a solitary omnivore
Source: Ecol Evol. 2018 Nov 23;8(23):12271–82. doi: 10.1002/ece3.4690 (PMC6303745; doi:10.1002/ece3.4690)
Supplement: Supplementary file 1 [file ECE3-8-12271-s001.docx]

Appendix

Table A. Median home range areas (km^2^), proportion of forest, and subsample size (n) for black bears *Ursus americanus* (2008-2017) in Michigan, Missouri, and Mississippi, USA.

| State | Sex | Annual Area | Proportion of forest | Season | Seasonal Area | Proportion of forest |
| --- | --- | --- | --- | --- | --- | --- |
| Michigan | F | 16.4 (15) |  | Fall | 5.9 (14) | 0.90 |
|  |  |  | 0.87 | Spring | 7.5 (21) | 0.91 |
|  |  |  |  | Summer | 15.8 (27) | 0.85 |
|  | M | 96.6 (8) |  | Fall | 9.2 (7) | 0.83 |
|  |  |  | 0.83 | Spring | 60.3 (11) | 0.84 |
|  |  |  |  | Summer | 83.3 (29) | 0.83 |
| Missouri | F | 34.6 (25) |  | Fall | 16.4 (74) | 0.94 |
|  |  |  | 0.93 | Spring | 16.0 (43) | 0.95 |
|  |  |  |  | Summer | 29.5 (77) | 0.91 |
|  | M | 182.9 (6) |  | Fall | 35.4 (24) | 0.92 |
|  |  |  | 0.89 | Spring | 102.8 (10) | 0.87 |
|  |  |  |  | Summer | 91.9 (31) | 0.87 |
| Mississippi | F | 16.7 (36) |  | Fall | 13.9 (51) | 0.87 |
|  |  |  | 0.89 | Spring | 6.9 (41) | 0.96 |
|  |  |  |  | Summer | 15.9 (53) | 0.88 |
|  | M | 60.0 (7) |  | Fall | 41.1 (15) | 0.67 |
|  |  |  | 0.69 | Spring | 20.6 (11) | 0.72 |
|  |  |  |  | Summer | 41.4 (10) | 0.69 |

Table B. Competing (< 2 AICc) generalized linear mixed models for annual home range size variation of black bears in Michigan, Missouri, and Mississippi (2008-2017). Best fit model (see CH3 results) in bold.

| (Int) | Edge density | Proportion of Forest | Sex | State | Sex:State | df | logLik | AICc | delta | weight | R2c | R2m |
| --- | --- | --- | --- | --- | --- | --- | --- | --- | --- | --- | --- | --- |
| 1.35 | -0.11 | -0.11 | + | + | + | 10 | -2.959 | 28.5 | 0 | 0.27 | 0.55 | 0.85 |
| **1.37** | **-0.10** |  | **+** | + |  | **7** | **-7.01** | **29.3** | **0.8** | **0.19** | **0.53** | **0.85** |
| 1.39 | -0.11 | -0.05 | + | + |  | 8 | -6.404 | 30.4 | 1.97 | 0.10 | 0.53 | 0.86 |

Table C. Competing (< 2 AICc) generalized linear mixed models for seasonal home range size variation of black bears in Michigan, Missouri, and Mississippi (2008-2017). SSn=season, Stt=state. Prop forest..= proportion of forest. Best fit model (see CH3 results) in bold.

| Int | Edge den. | Mean NDVI | Prop. Forest | Ssn | Sex | Stt | Ssn:sex | Ssn:Stt | Sex:Stt | df | logLik | AICc | delt | weight | R2c | R2m |
| --- | --- | --- | --- | --- | --- | --- | --- | --- | --- | --- | --- | --- | --- | --- | --- | --- |
| **1.11** | **-0.18** |  | **-0.06** | **+** | **+** | **+** | **+** | **+** |  | **16** | **-137.86** | **308.8** | **0** | **0.37** | **0.55** | **0.68** |
| 1.06 | -0.18 |  | -0.07 | + | + | + | + | + | + | 18 | -136.26 | 309.9 | 1.09 | 0.21 | 0.55 | 0.69 |
| 1.11 | -0.18 | -0.01 | -0.06 | + | + | + | + | + |  | 17 | -137.80 | 310.8 | 2.03 | 0.13 | 0.55 | 0.68 |
